# Application of Lavender-Oil Microcapsules to Functionalized PET Fibers

**DOI:** 10.3390/polym15040917

**Published:** 2023-02-11

**Authors:** Rita de Cássia Siqueira Curto Valle, José Alexandre Borges Valle, Fabricio Maestá Bezerra, Jeferson Correia, Cristiane da Costa, Meritxell Martí, Luisa Coderch, Arianne López, Manuel J. Lis Arias

**Affiliations:** 1 Department Textile Engineering, Federal University of Santa Caterina, Blumenau 89036-002, SC, Brazil; 2 Textile Engineering (COENT), Federal Technological University of Paraná, Apucarana 86812-460, PR, Brazil; 3 Institute of Advanced Chemistry of Catalonia (IQAC-CSIC), 08034 Barcelona, Spain; 4 Surface Science Laboratory, INTEXTER-UPC, 08222 Terrassa, Spain

**Keywords:** ozone, plasma, microcapsules, essential oils, drug delivery, surface functionalization

## Abstract

Surface treatments for textile substrates have received significant attention from researchers around the world. Ozone and plasma treatments trigger a series of surface alterations in textile substrates that can improve the anchoring of other molecules or particles on these substrates. This work aims to evaluate the effect of ozone and plasma treatments on the impregnation of polymeric microcapsules containing lavender oil in polyester fabrics (PES). Microcapsules with walls of chitosan and gum arabic were prepared by complex coacervation and impregnated in PES, plasma-treated PES, and ozone-treated PES by padding. The microcapsules were characterized for their size and morphology and the surface-treated PES was evaluated by FTIR, TGA, SEM, and lavender release. The microcapsules were spherical in shape, with smooth surfaces. The FTIR analyses of the textile substrates with microcapsules showed bands referring to the polymers of the microcapsules, but not to the lavender; this was most likely because the smooth surface of the outer wall did not retain the lavender. The mass loss and the degradation temperatures measured by TGA were similar for all the ozone-treated and plasma-treated polyester samples. In the SEM images, spherical microcapsules and the impregnation of the microcapsules of larger sizes were perceived. Through the lavender release, it was observed that the plasma and ozone treatments interfered both with the amount of lavender delivered and with the control of the delivery.

## 1. Introduction

Textile-substrate functionalization is one of the most important developments to be conducted by academic or commercial researchers [1]. There is a wide range of motivations behind studies with micro/nanocapsules to produce attributes that involve the controlled transfer of active compounds under certain conditions [2]. This technology protects encapsulated substances against external factors, providing the durability of the active principle and the stability of the properties of the textiles, as well as adding value to the product [3,4]. The global pandemic crisis caused by COVID-19 drew attention to the necessity of human protection and led to a search for new ways of reducing virus transfer. In this context, masks and other textiles were demonstrated to be among the most effective methods for reducing the transmission of infectious diseases. 

Currently, polyester fiber is the most widely used method because it shows many advantages, such as good mechanical properties, moisture resistance, resistance to temperature and light, and good chemical resistance to solutions of acids and bases at room temperature [5]. However, it has high crystallinity, low surface energy, and poor wettability [6], which make it difficult to create functionalities by anchorage with conventionally available chemicals [7]. The modification of the surface layers (SLs) of polymers is one of the ways to achieve the desired surface properties [8]. In the SLs of synthetic polymers, there are low-molecular-weight macromolecules (oligomers), and different materials are located more deeply [9]. Deshmukh and Bath [10] presented many surface-modification techniques, such as wet chemical processing, mechanical abrasion, flame treatment, enzymatic-surface modification, sonication, and plasma processing, to increase the adhesion capacity due to changes in the SL chemical composition by generating new surface-functional groups, which may lead to the formation of chemical bonds between atoms of the SL and molecules of the substance to be deposited on it. The plasma treatment of polymer surfaces not only causes a modification during the plasma exposure, but also leaves active sites at the surfaces, which are subject to post-reactions; this is also called aging [11]. The ozonization technique is often used in polymer-surface modification due to its easy procedure and low cost [12]. Ozone is a potent oxidizing agent that may react with surface polymers [13] and some carbonyl groups, and carboxyl groups appear on the polymer chains [14]. The surface wettability may be increased in polymers exposed to plasma or ozone, and both techniques have been used for the enhancement of interfacial adhesion [12]. In addition, the use of active oxygen (produced by plasma reaction or added directly) treatment is recognized as a safe treating method [14].

Studies have been conducted on the use of microcapsules with different polymers to protect and release many kinds of active principles and for many applications, such as cosmetics [15], insect repellents [16,17], personal care [4], and medical and pharmaceutical treatments [18,19]. In comparison with synthetic polymers, biopolymers used as microcapsule shells have been preferred due to their biodegradability, renewability, nontoxicity, safety, and biocompatibility [20,21]. Many natural polymers can be applied. Chitosan, due to its polycationic nature in acid media, can associate with anionic chemical groups to form interesting complexes [22]. In addition, gum arabic is a polysaccharide with high water solubility, good emulsification ability, and film-forming properties that protect the sensitive core material [23].

Essential oils (EOs) are liquid products present in plants and can be defined as complex natural mixtures of volatile secondary lipophilic metabolites [24]. They have many interesting applications as sources of bioactive compounds and have emerged as effective antimicrobials. They are generally recognized as safe (GRAS) agents [25] and non-toxic to the environment, and they offer the advantage that microorganisms cannot acquire resistance against them due to the presence of a large variety of constituents [26]. Lavender oil has in its composition some aromatic compounds that provide a pleasant fragrance and antimicrobial protection [27], with more than 100 different molecules [28]. This oil has been closely studied for use in textiles, with ca. 80 documents with “microcapsule and textile and lavender” as keywords, according to Scopus (2022). These include studies of the oil’s microencapsulation process [29,30,31,32], textile applications [4,33], and release [32]. 

Based on this, the objective of this study is to evaluate the functionalization of modified polyester fabric with gum arabic/chitosan microcapsules containing lavender oil. For this study, polyester non- and modified polyester with CD plasma and ozone were used.

## 2. Materials and Methods

### 2.1. Materials

Plain woven PET (Style 700-480), polyester poplin, 150 g/m^2^, lavender oil, chitosan, Tween 20, and gum arabic were kindly supplied by CARINSA Group (Spain).

### 2.2. Chemical Modification of Samples

The CD plasma pre-treatments were performed with the Corona plus Type TF-415 (Vetaphone, Kolding, Denmark), with a constant gap between the electrode and roll (4 mm). The sample was passed once, twice or five times through the discharge area at treatment powers 200 W, 400 W, and 800 W.

### 2.3. Polyester Modification by O_3_

The treatment of substrates with ozone was performed using the procedure established by Gabardo et al. [33]. In summary, ozone modification was performed using UV-SURF X4 (UV-Consulting Peschl España, Spain) equipment, with 17 W of power, and an emission spectrum varying from 185 to 254 nm. Polyester samples of 10 cm × 20 cm were inserted into the equipment’s chamber and exposed for 20, 30, and 45 min to ozone production by low-pressure mercury lamps.

### 2.4. Preparation and Application of Lavender-Oil Microcapsules

The microcapsules were produced by complex coacervation between chitosan (1% *w*/*v*), gum Arabic (2% *w*/*v*), and lavender oil in emulsion 2% (*v*/*v*). First, the biopolymer solution was prepared separately. The chitosan was dissolved in acid acetic, 0.1 M, with magnetic agitation for 15 min at 40 °C. Gum Arabic solution was prepared in deionized water under stirring conditions at 40 °C to reach complete dissolution. The lavender oil (2%) was stabilized in aqueous media with Tween 20 (2%) under stirring at 3000 rpm for 1 min (IKA, T-25). 

The process was conducted by mixing the chitosan and lavender-oil solutions to form the first polymer layer and activate it. After adjusting the pH to 3.5 with chloride acid 1 M, the gum Arabic solution was added to the construction of the second layer. Once the mixture was achieved under magnetic agitation, it was emulsified for 1 min at a stirring rate of 8000 rpm (Ultraturrax T-25, IKA, Staufen, Germany). Finally, the temperature of the mixture was reduced to 5 °C, and the cross-linking agent, the 2 mL of tannic acid (10% *v*/*v*), was placed in 100 mL of solution and kept at a constant temperature (5 °C) under magnetic agitation for 3 h. Particle-size distribution of microspheres was carried out with a Malvern Nanosizer (Mastersizer-2000, UK). The average size was determined with the laser-particle analyzer at 25 °C.

### 2.5. Polyester-Fabric Functionalization

The microcapsules were immobilized onto fabric by a conventional pad–dry–cure process using citric acid as a crosslinking agent [34]. A solution was prepared with each of the samples containing 10% (*w*/*v*) of the dissolution of the microcapsules, 3% (*w*/*v*) citric acid, and 3% (*w*/*v*) monobasic sodium phosphate monohydrate (catalyst). The textiles were immersed in the aforementioned finishing liquid (bath ratio = 1:20) for 10 min and, with the help of a foulard, the samples were impregnated at a pressure between rolls of 1 bar, producing a wet pickup of approximately 90%. Drying was carried out at 80 °C for 3 min.

### 2.6. Evaluation of Modified Fabric

The FTIR spectrophotometric measurements were conducted using Shimadzu—Model FTIR-8300 in the spectral region of 4000 to 500 cm^−1^ to verify the interactions of the polymers of microcapsules and textiles. Mettler-Toledo Thermogravimeter (TGA) was used to evaluate the microstructural changes of functionalized fabrics (Mettler-Toledo, Columbus, OH, USA). The test was performed at a speed of 10 °C min^−1^, in the temperature range of 30 °C to 550 °C, in a nitrogen atmosphere with a flow of 50 mL min^−1^. 

### 2.7. In Vitro Release Behavior

The release of microencapsulated lavender oil incorporated into the textiles was performed using the technique introduced by Ghaheh et al. [35] and Carreras et al. [36]. Samples of polyester fabrics measuring 2 cm × 2 cm were placed in Erlenmeyer with 24 mL of distilled water and 1 mL of ethanol (WEt) thermo-stabilized at 37.0 ± 0.5 °C in a static bath. Aliquots of 1.2 mL were extracted at predetermined times for 300 min. Their absorbance was determined by spectroscopy (Shimadzu—UV-240LPC) in the ultraviolet region, between 700 and 200 nm. (oil), corresponding to the maximum peak of absorption. A calibration curve for the oil concentration was constructed to determine the amount of active-ingredient lavender compound released into the medium from the microcapsules. After each sample withdrawal, the same volume of fresh WEt was added to the receptor medium and kept constant in the receptor solution during the experiment [36].

### 2.8. Scanning Electron and Optical Microscopy

The morphology, size, and polydispersibility of the microcapsules were recorded using an optical microscope, Olympus BX43F, coupled to a Schott KL300 LED digital camera and Micron Measurement video-recording software. Superficial morphological and topographical analyses of the polyester fabrics were carried out with scanning electron microscope JEOL, JS-5610, and the gold overlay was made with the SCD 005, Bal-Tec equipment. 

## 3. Results

### 3.1. Microcapsules

**Figure 1:**
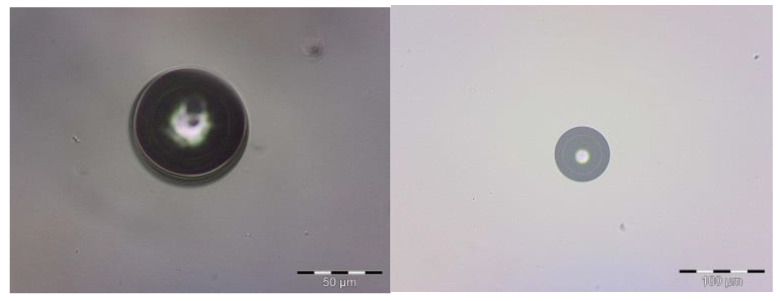
Optical micrographs of chitosan microcapsules.

The microcapsules obtained presented a circular form with minimal aggregation, as shown in Figure 1. This result is similar to that found by Ocak et al. [37], who microencapsulated lavender oil through the complex coacervation technique, involving microcapsules with only one nucleus coated by a polymer. The average diameter of the microcapsules was 15.62 µm; this was obtained with zeta sizer equipment. When it was possible to observe bigger particles, the optical microscopy revealed that the lavender oil was in the center. The optical-microscopy images could be used as a model to determine the formation of layers. The difference in coloration indicated that the surfactant was on the inner edge of the shell. Two layers were visibly formatted; the internal layer was composed of chitosan and the external layer was composed of gum Arabic. Between the polymers was water that migrated during the reticulation process.

**Figure 2:**
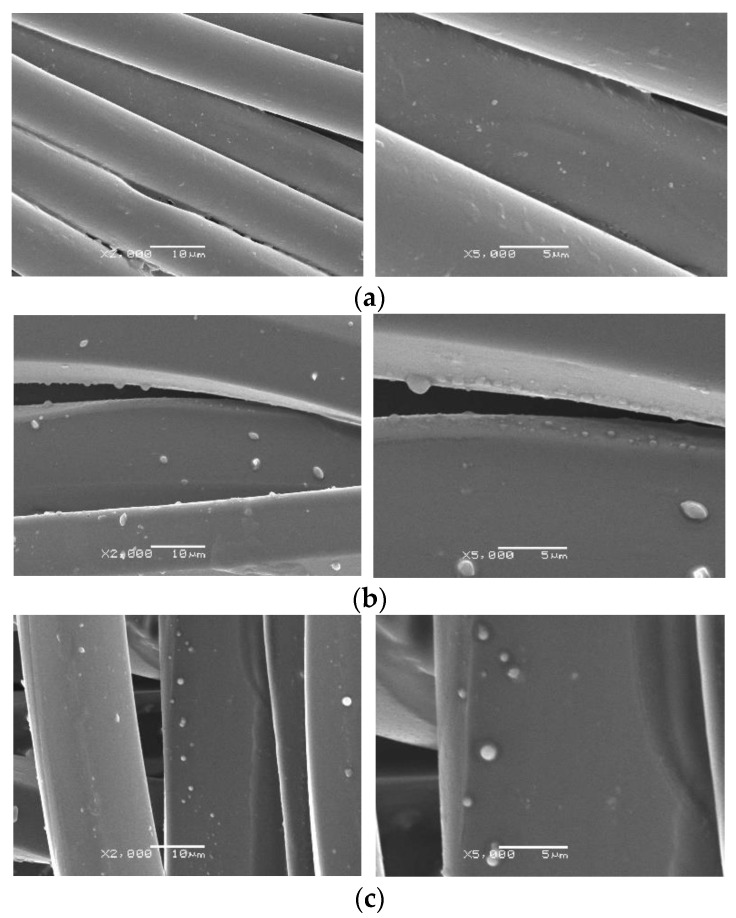
Morphology of polyester-impregnated samples: (**a**) untreated polyester, (**b**) polyester treated with plasma, (**c**) polyester treated with ozone. Magnification of 2000 and 5000x.

The spherical shapes of the produced microcapsules are also presented in the SEM micrographs obtained for the polyester fabrics impregnated with microcapsules (Figure 2). Comparing the untreated and plasma- or ozone-treated samples, a higher amount of microcapsules was impregnated in the polyester fabrics with the plasma or ozone treatments. In addition, it was verified that the particles were dispersed on the surface of the fiber, without clusters. The generation of physical voids through the plasma treatment increased the absorption capability of the textile substrate, mainly particles of smaller size, as observed in the micrographs; the largest sizes were around 2 µm. The effect of ozone or plasma treatment may cause slight erosion in the surfaces of fibers, favoring the adhesion of small particles. In the case of plasma, Peran and Razic [38] and Ayesh et al. [39] stated that the effect of the plasma is dependent of the conditions under which the fiber is exposed in the treatment. Similarly, Lou et al. [40] demonstrated that ozone affects the surface morphology of polyester fibers. In this study, the ability of the polyester fiber treated with plasma and ozone to adhere to the biopolymer was very similar, and the effect was related to the particle size.

### 3.2. FTIR

**Figure 3:**
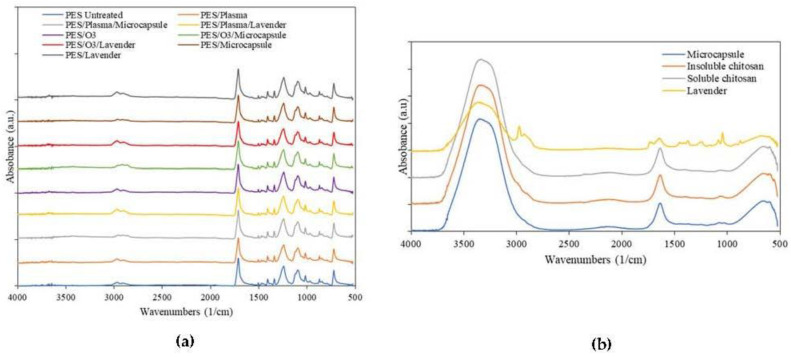
FTIR spectra of the (**a**) polyester treated and untreated with and without microcapsules and (**b**) microcapsules and compounds isolated from the constitution.

The FTIR-ATR spectra of the polyester samples are shown in Figure 3. The bands present in the spectrum were identified and correlated with the chemical structure of the polyester fiber [41]. The spectra of the samples, in Figure 3a, present bands in the region of 720–870 cm^−1^, which refer to angular deformation (CH_2_)_n_, the vibrations of adjacent aromatic hydrogens, and the vibration of the aromatic ring of the benzene functional group [42,43]. Other important bands present in the spectra were the 1241 cm^−1^ strong stretching of the C-O group of unsaturated and aromatic ester, 1338 cm^−1^ CH_2_ bending-type angular deformation, 1407–1471 stretching of the C-O group and deformation of the OH group, 1504–1577 aromatic skeleton vibration with C=C stretch, and 1713 stretching the C=O carbonyl group of carboxylic acid groups. As can be seen, the surface modifications did not cause changes when comparing the spectrograms. This was because IR spectroscopy is performed by light absorption, with which it is possible to observe functional groups with large changes in the dipoles, while functional groups with weak dipole changes or with a high degree of symmetry, as in the chemical structure of polyester, do not change. Similar results were presented by Gabardo et al. [33].

Regarding the materials, the oscillations in the region of 3317 cm^−1^ referred to hydrogen -OH groups [43], and the carbonyl bands were observed at 1635 cm^−1^ [44] and 1240 cm^−1^ for the NH bend [45]. The bands present in the spectra of capsules were the same as those shown by the chitosan. This occurred because the wall polymer used to form the microcapsules was made of chitosan. 

The main bands of the lavender spectrum were as follows. In the spectral region of 2800–3400 cm^−1^, the bands at 2937 and 2975 cm^−1^ represented –CH_2_- stretching, while the IR band at 3380 cm^−1^ was attributed to O–H stretching vibrations [46]. Another band that appeared in the spectrum was in the region of 1734 cm^−1^, the band of C=O stretching [47], 1227 cm^−1^ C–O stretching vibrations, and 1365, 1430, and 1475 cm^−1^ C–H bending vibrations [48].

In all the samples in the first group, (a) the predominant peaks were, as expected, the signals corresponding to the main substrate. The modifications on the surface were not strong enough to modify the principal groups of the PET substrate. The remaining components of the system (b) showed that, except for the essential-oil spectrum, there are many similarities between the chitosan and the external layer of the microcapsule. The convolution of signals corresponding to -NH and -OH chemical groups made it impossible possible to establish, with enough accuracy, the expected molecular interactions between them in the outer layer of the microcapsule. 

The effect of plasma- and polyester-ozone pretreatments was evaluated with FTIR, TGA, and electronic-microscopy analysis. Neither pretreatment demonstrated molecular modification into the fabric. Similar results were presented by Nowak et al. [42], in whose study the structural changes of the polymer were not significantly affected. 

### 3.3. Thermal Analysis 

**Figure 4:**
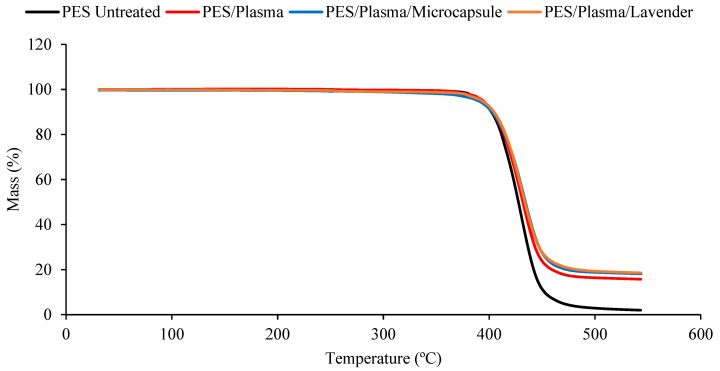
The TGA measurements of the untreated polyester and plasma-treated samples, impregnated with lavender essential oil or microcapsules. Untreated polyester (black), plasma-treated polyester (red), plasma-treated polyester with microcapsules (blue), and plasma-treated polyester with the lavender essential oil (brown).

**Figure 5:**
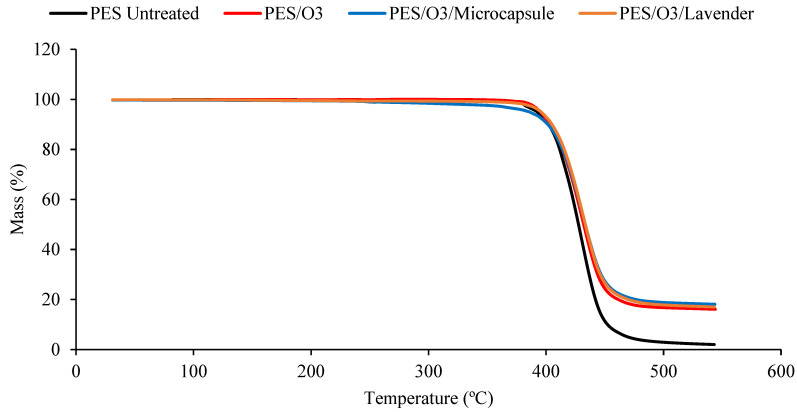
The TGA measurements of the untreated polyester and ozone-treated samples, impregnated with lavender essential oil or microcapsules. Untreated polyester (black), ozone-treated polyester (red), ozone-treated polyester with microcapsules (blue) and ozone-treated polyester with the lavender essential oil (brown).

**Figure 6:**
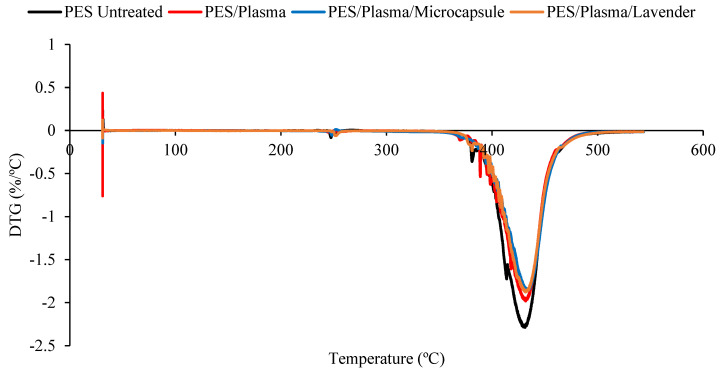
The DTG comparison of the untreated polyester and plasma-treated samples, impregnated with lavender essential oil or microcapsules.. Untreated polyester (black), plasma-treated polyester (red), plasma-treated polyester with microcapsules (blue), and plasma-treated polyester with the lavender essential oil (brown).

**Figure 7:**
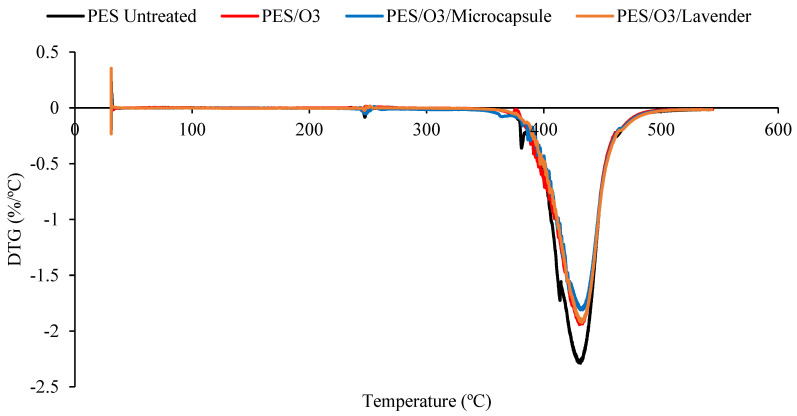
The DTG comparison of the untreated polyester and ozone treated samples, impregnated with lavender essential oil or microcapsules. Untreated polyester (black), ozone-treated polyester (red), ozone-treated polyester with microcapsules (blue) and ozone-treated polyester with the lavender essential oil (brown).

The thermal stability of the untreated polyester and the polyester fabrics treated with plasma or ozone, with or without microcap impregnation, was determined using thermogravimetric analysis (TGA; Figure 4 and Figure 5). The DTG curves, obtained from TGA, are shown in Figure 6 and Figure 7. All the samples presented a small peak—a small mass loss—at nearly 250 °C, and a significant mass loss between 350 and 470 °C. The observed mass loss was due to the thermal degradation of the polyester fabrics, which was also shown by Manasoglu et al. [48]. 

The decomposition temperature ranges, the DTG peak temperature, and the mass loss are presented in Table 1. All the samples presented degradation in the same temperature interval, from 350 to 470 °C. In this range, the mass loss was similar for all the treated samples. It was possible to verify that all the treated polyester fabrics (ozone- or plasma-treated) presented a final residue superior to that of the untreated polyester, showing that the treatments changed the characteristics of the textile material. The principal results are shown in Table 1.

Although the differences caused by the treatments were slight, several remarkable facts need to be considered. The T_max_ reached by the samples in the principal thermal-decomposition range, except in the case of the polyester with microcapsules, tended to increase. The lack of affinity for polyester fiber, without treatment, and the external shell (gum arabic, anionic character) tended to decrease this temperature because of the earlier decomposition of the gum-arabic shell. In the remaining cases, the surface treatments increased the ability of the surface to allocate either the microcapsules or the essential oil, which was translated into an increase in the maximum temperature of the aforementioned thermal decomposition. There were more molecular interactions between the treated surface and the active principles incorporated into it. 

The interactions in the plasma-treated samples increased towards 367 °C, while in the case of the ozone treatment, the resulting surface reached a lower level of interactions and stayed at 363 °C. In every case, the intensity of the interactions was higher between the treated surfaces and the external shells of microcapsules. This is a very interesting result because, to a certain extent, it demonstrates that the essential oil presents a higher power of retention when it is allocated inside the microcapsule than when it is applied without it. In this way, an effect of prolonged volatility of the microcapsule is produced, which is harmless to the textile material. This was also shown in the work of Yingngam et al. [16].

### 3.4. Lavender Release

**Figure 8:**
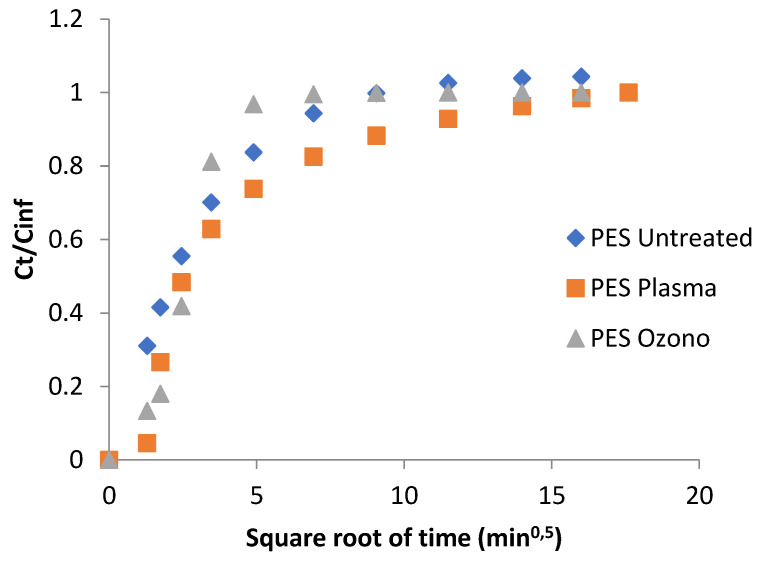
Relative release of lavender oil, for the different square roots of time, from the microcapsules impregnated in polyester fabrics.

Ultraviolet–visible spectroscopy is used to identify and quantify compounds through electronic transitions induced by the absorbed radiation by the molecule pela in a specific region of the spectra. In this case, the UV-VIS was used to study the diffusion phenomena of the lavender oil in water at 37 °C from the microcapsules produced for complex coacervation. The behavior of the release of the lavender oil (Figure 8) shows that the polyester treated with ozone liberated more oil. This phenomenon was potentially due the polarization of the ozone on the surface of the polyester increasing the affinity for the microcapsules. This affinity involves a major adhesion of the microcapsules and, as a consequence, an increase in the availability of oil for liberation. On the other hand, the effect of the plasma has a relationship with the formation of pores without chemistry changes, and does not interfere with the affinity of the microcapsule, whose external layer is a polar polymer. The delay in the oil release from the polyester treated with plasma can also be related to the formation of pores that could have supported the microcapsules and hampered the removal of the oil. The complete release of the lavender oil to the medium lasted around 80 min for the polyester without treatment. A similar result was presented in the work by Bezerra et al. [49], who microencapsulated an essential oil through complex coacervation and applied the formed microcapsule in polyester fabric, also obtaining release balance in approximately 80 min. However, when the surface modification was performed, the values found were 250 min and 25 min for the polyesters treated with plasma and ozone, respectively.

As can be seen in the figure, the behavior can be divided into three different regions. In the first part, what can be said to comprise short times (around 4 min), when the bath remained nearly infinite, the slope was related to the pure diffusion from the tissue. In this range of time, the rate of delivery of the essential oil was higher in the PES without treatment than that observed in the ozone- and plasma-treated samples. In the case of the plasma treatment, the rate was the lowest, while the plasma case showed an intermediate value. This short-term mechanism corroborates the results obtained from the DTGA/TGA (Table 1). The retention power of microcapsules is higher when PES is treated with plasma than when it is treated with ozone. The pre-treatments can cause roughness on fiber surfaces and can improve some proprieties in polyester molecules [39], which can change the affinity with lavender oil, which in turn can delay the release of the active principle. 

In a second range of values, comprising the intermediate times (4–100 min), the first amount of active principle delivered began to acquire a certain importance and included the greater or weaker affinity of the essential oil for water. As demonstrated previously, the essential oil showed a hydrophobic character and, therefore, the slope corresponding to this range of times showed crossing lines between the plasma- and ozone-treated samples, while the PES remained in a state of continuous delivery. After this second step in the delivery of the essential oil, the last part of the graph shows the behavior over a very long time, near to equilibrium. Here, all the samples reached the maximum possible amount of delivery at 225 min.

Over the whole range of the tested times, the plasma-treated samples show more controlled behavior, following the first-time tendency toward a lower rate of delivery. 

## 4. Conclusions

In the application of essential oils to textile substrates, the influence of the substrate has been poorly studied. In this case, using lavender oil as a tracer, we demonstrated that there is a strong influence on the surface of the substrate, especially when dealing with non-polar substrates. The plasma-treated and ozone-treated samples presented homogenous distribution particles, and it was verified that the preferential adhesion of smaller microcapsules was probably due to the pore sizes formed on the polyester fiber through the actions of the ozone and plasma action. 

The external character of the shell in the microcapsules influenced not only the number of microcapsules to be applied but also how the active principle was delivered. These results can be assigned to property changes in the polyester molecules of the fiber surface, which changes the affinity of the oil with the polyester. Previous treatments of the surface, plasma, or ozone can help to gain power retention and to control the rate of delivery at different times. 

The influence can be detected using FTIR and TGA when dealing with the structural changes occurring on the surface, but it is also clearly detectable when drug-delivery kinetics are studied. Based on these results, the combination of microcapsules and modified surfaces can be used in a wide range of applications in the field of biofunctional textiles.

## Figures and Tables

**Table 1:** The TGA data of samples: initial degradation temperature (T_i_), major degradation temperature (T_max_), final degradation temperature (T_f_), and mass loss of polyester fabric.

Sample	T_onset_	T_max_	T_offset_	Mass Loss (%)
Untreated Polyester (PES)	351.22	430.74	466.39	98.01
PES/Microcapsule	349.18	429.95	467.09	82.17
PES/LAV	356.91	433.50	466.30	85.09
PES/PLASMA	367.13	431.24	466.09	84.26
PES/PLASMA/Microcapsule	367.13	433.30	461.35	81.87
PES/PLASMA/LAVENDER	364.76	433.30	466.52	81.43
PES/O3	363.14	432.44	467.08	83.91
PES/O3/Microcapsule	363.14	436.24	467.92	81.91
PES/O3/LAVENDER	360.18	432.44	466.86	82.99

## Data Availability

The data that support the findings of this study are available on request from the corresponding author.
